# Human type H vessels are a sensitive biomarker of bone mass

**DOI:** 10.1038/cddis.2017.36

**Published:** 2017-05-04

**Authors:** Liang Wang, Fei Zhou, Peng Zhang, Hongzhen Wang, Zhipeng Qu, Peng Jia, Zhe Yao, Guangsi Shen, Guangfei Li, Guoyang Zhao, Jian Li, Yongtao Mao, Zonggang Xie, Wei Xu, Youjia Xu, Ying Xu

**Affiliations:** 1 Department of Orthopaedics, the Second Affiliated Hospital of Soochow University, Suzhou 215004, China; 2 Cam-Su Genomic Resource Center, Soochow University, Suzhou 215123, China; 3 Department of Orthopedics, the First Peoples’ Hospital of Kunshan, Suzhou 215300, China; 4 Department of Orthopedics, Affiliated Hospital of Jiangsu University, Zhenjiang 212001, China

## Abstract

Vascularization is fundamental for bone formation and bone tissue homeostasis. However, in human subjects, a direct molecular relationship has not been identified between angiogenesis and agents that promote bone disease or factors related to age. Osteopenia is a condition in which bone mineral density is lower than normal, and it represents a sign of normal aging. Here we tested whether the type H vessel, which was recently identified as strongly positive for CD31 and Endomucin (CD31^hi^Emcn^hi^) in mice, is an important indicator of aging and osteopenia in human subjects. We found that age-dependent losses of type H vessels in human bone sections conform to the observations in aged mice. The abundance of human type H vessels and osteoprogenitors may be relevant to changes in the skeletal microarchitecture and advanced osteopenia. Furthermore, ovariectomized mice, a widely used model for postmenopausal osteoporosis, exhibited significantly reduced type H vessels accompanied by reduced osteoprogenitors, which is consistent with impaired bone microarchitecture and osteoporosis, suggesting that this feature is an indicator of bone mass independent of aging. More importantly, administration of desferrioxamine led to significantly increased bone mass via enhanced angiogenesis and increased type H vessels in ovariectomized mice. Altogether, these data represent a novel finding that type H vessels are regulated in aged and osteopenia subjects. The abundance of human type H vessels is an early marker of bone loss and represents a potential target for improving bone quality via the induction of type H vessels.

The loss of bone mass experienced by many postmenopausal women and elderly men has the potential to weaken bones and increase the likelihood of bone fracture and could lead to the onset of osteoporosis.^1^ Studies in recent years have greatly expanded our understanding of bone formation in mice, and research has been performed to evaluate osteoporosis associated with inflammatory states;^2, 3^ ATF4,^4, 5^ WNT and Notch signaling in the regulation of the function and differentiation of osteoblasts;^6, 7, 8, 9, 10^ and cell behavior.^11^ These studies have expanded the scope of potential molecular targets for anti-osteoporosis therapy. Clinical practices have probed the value of molecular targets for osteoporosis treatment, including the RANKL–RANK pathway.^12^ These basic studies suggest that identifying the molecular pathways that regulate the coupling process may lead to the development of new therapeutics capable of simultaneously preventing bone loss and inducing new bone formation.

Blood vessels present detrimental and beneficial effects on bone growth and hemostasis. However, blood vessels also serve as a source of mesenchymal stem cells, which can be induced to differentiate into osteoblasts for new bone formation.^13, 14^ The slow penetration of host blood vessels in large engineered bone tissue grafts impedes bone construction;^15^ thus, angiogenesis plays a pivotal role in bone development and bone fracture repair.^16, 17^ Angiogenic–osteogenic coupling is crucial for bone homeostasis, and it is tightly regulated by a specific capillary subtype in bone that is known as a type H vessel in mice.^18^ These type H vessels have been identified in specific locations, mainly in the metaphysis near the growth plate and in the endosteum. These structures have been shown to mediate the growth of the bone vasculature, maintain perivascular osteoprogenitors, and couple angiogenesis to osteogenesis.^18, 19^ The endothelial cells of the type H vessel exhibited distinct molecular properties and are strongly positive for the endothelial cell surface markers CD31 and Endomucin (CD31^hi^Emcn^hi^) as assessed by immunofluorescence staining. Interestingly, osteoprogenitor cells (Osterix^+^), which further differentiate into osteoblasts and osteocytes,^20^ are located directly adjacent to type H vessels, which suggests that reprogramming endothelial cells into type H endothelial cells may be beneficial for the treatment of osteoporosis or the promotion of bone fracture healing. Moreover, platelet-derived growth factor-BB, which is secreted by preosteoclasts, increases the number of type H vessels and stimulates osteogenesis in ovariectomized mice.^19, 21^ These studies from murine models have led to the question of whether improved type H vessels might have the potential for use as a therapeutic for the improvement of osteogenesis in elderly people and postmenopausal women. However, considerable differences in bone anatomy, macrostructure, and microstructure occur between mice and humans, and information on type H vessels in human subjects remains elusive. In addition, it is unclear whether reductions in type H vessels are an independent effect of decreased bone mass or caused by age-related factors. However, both of these scenarios may be possible, and despite recent promising findings for type H vessels, this information remains poorly understood.

In this study, we confirmed that type H vessels and osteoprogenitors in specific locations of murine tibiae and femurs declined in age-dependent mice models, and we also observed that type H vessels coupled with osteoprogenitors are present in the human proximal femur near the greater trochanter and showed that these vessels were markedly reduced in elderly people. A comparison of the control, osteopenia, and osteoporosis groups showed that human type H vessels were more closely correlated with bone loss and the abundance of type H vessels decreased in osteopenic and osteoprotic subjects. Thus, our results strongly suggest that human type H vessels are an early indicator of bone loss.

## Results

### Age-dependent decline of type H vessels and osteoprogenitor cells in mouse tibiae and femurs

**Figure 1:**
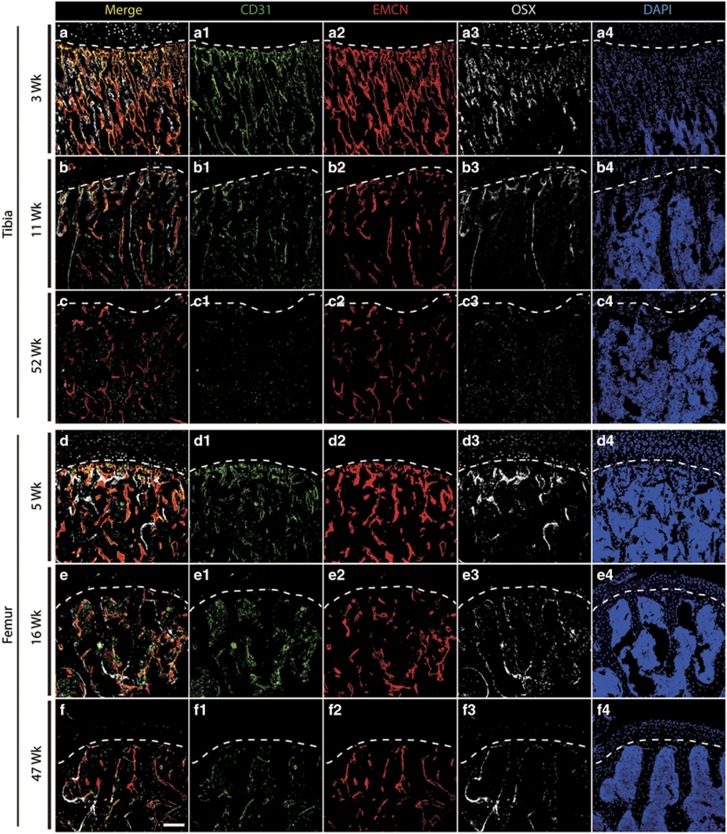
Age series of mouse tibia and femur immunostaining. (**a**–**c**) Tibia immunostaining for CD31 (green), Endomucin (red), Osterix (gray), and DAPI (blue) for juvenile (3 weeks, **a**), adult (11 weeks, **b**), and aged (52 weeks, **c**) mice. (**d**–**f**), Femur (proximal region) immunostaining for CD31 (green), Endomucin (red), Osterix (gray), and DAPI (blue) for juvenile (5 weeks, **d**), adult (16 weeks, **e**), and aged (47 weeks, **f**) mice. Line in each panel indicates the boundary of metaphysis and growth plate. Scale bar: 100 *μ*m

The type H vessel has been identified in bone structures and is positive for the vascular markers CD31 and Endomucin, and its involvement in the coupling between angiogenesis and osteogenesis has been reported.^18^ Immunostaining confirmed that an abundant amount of type H vessels and associated osteoprogenitors (Osterix^+^) are present in juvenile mouse tibiae, especially at metaphyseal and periosteal regions (Supplementary Figure 1). We then examined aging-related changes in tibiae obtained from juvenile (3–5 weeks), adult (11–16 weeks), and aged (47–52 weeks) mice. Type H vessels and osteoprogenitors exhibited accelerated loss along with aging (Figures 1a–c), which is consistent with a previous report.^18^ To test the expression of type H vessels in human subjects in the subsequent experiments, we chose to investigate the distal and proximal metaphysis in mice because hip fracture is more common and human femur samples collected after surgery are readily available compared with tibia samples. The abundance of type H vessels and osteoprogenitors was obviously decreased in the proximal and distal metaphysis of the aged mouse femurs (Figures 1d–f and Supplementary Figure 2) compared with that of the juvenile and adult mice, which was expected. These results confirmed that type H vessels are a sensitive marker for age-related changes in murine bones.

### Reduction of type H vessels and osteoprogenitors in aged human subjects

**Figure 2:**
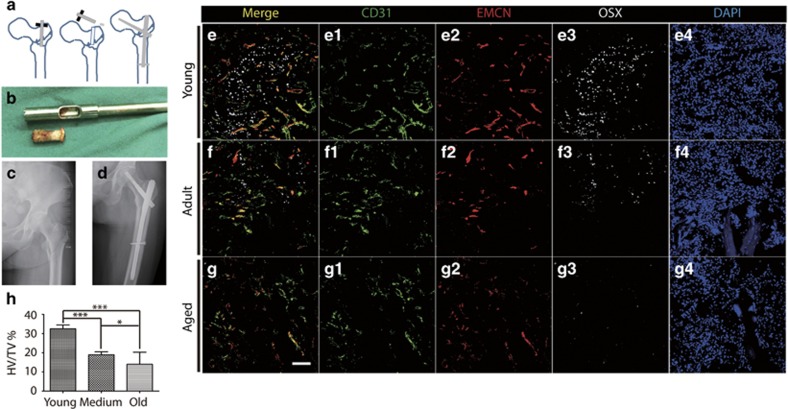
Decreased type H vessels in aged patients. (**a**), Schematic illustration of bone sample collection from patients during surgery. (**b**), Typical human bone sample collected from PFN surgery. (**c**,**d**), Pre- and post-surgical X-ray images of a typical patient. (**e**–**g**), Bone immunostaining for CD31 (green), Endomucin (red), Osterix (gray), and DAPI (blue) for young (**e**), adult (**f**) and aged (**g**) patients. (**h**) Quantification of type H vessel percentage (HV/TV%) in young (*N*=7), adult (*N*=8), and aged patients (*N*=24). Graphs represent mean±S.D.; *P<0.05; ***P<0.0001; Scale bar: 100 *μ*m

To test whether type H vessels are also present in human bone tissues, bone samples were obtained during proximal femoral nail (PFN) surgery using a specific device (Figures 2a and b). Preoperative and postoperative X-rays images were collected for all patients (Figures 2c and d) to visualize the bone structures. Human bone samples were immediately processed after collection and subjected to immunofluorescence using antibodies against human CD31, Endomucin (Emcn), and Osterix (OSX). We found that the expression of CD31^+^Emcn^+^ type H vessels was enriched in the proximal femur near the greater trochanter and Osterix^+^ osteoprogenitors were surrounded by type H vessels in the young subjects, which implied a functional association (Figures 2e–g). Type H vessels and osteoprogenitors exhibited a remarkable dynamic pattern, including enrichment in young subjects (20–35 years, 7 individuals), decline in the adult subjects (45–59 years, 8 individuals), and near absence in elderly subjects (60–75 years, 24 individuals) (Figures 2e–g), which is consistent with the results found in mice (Figure 1).^18^ In general, the absolute values of the type H vessels in human samples varied depending on the batch and/or procedures. To overcome these problems before performing the quantitative analyses, we normalized the volume of the type H vessels (CD31^hi^Emcn^hi^) to the volume of the CD31- and/or Emcn-positive signals (total vessels), which is referred to as HV/TV, to avoid a large bias. The percentage of HV/TV was 32.46±0.76% in the young subjects, 18.93±0.55% in the adult subjects, and 13.93±1.29% in the elderly subjects. The differences among these groups were significant (*P*<0.0001) by one-way ANOVA (Figure 2h), indicating that the pronounced reduction in the abundance of type H vessels is associated with aging. These observations established the presence of human type H vessels coupled with osteoprogenitors in the proximal femur near the greater trochanter and showed that human type H vessels provide a reliable marker for bone aging.

### Synergetic reduction of type H vessels in osteopenia or/and osteoporotic subjects

**Figure 3:**
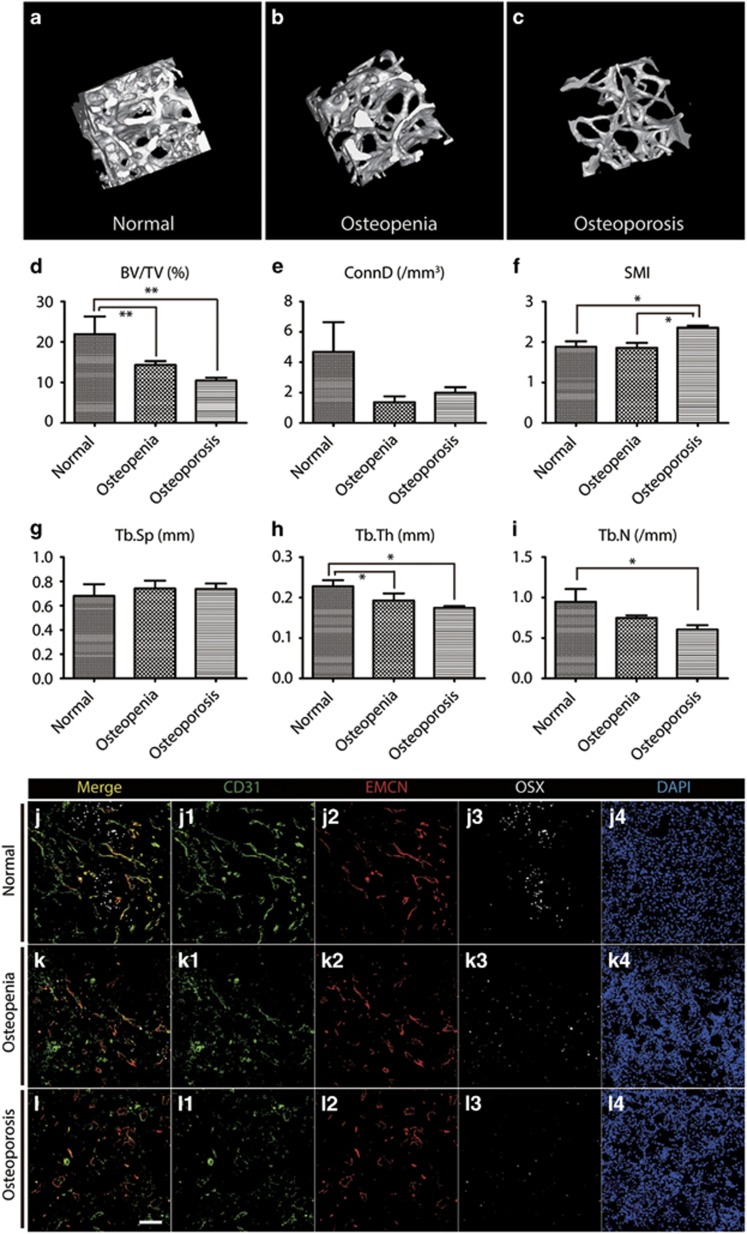
Synergistic decrease of type H vessel and bone mineral density in human bone. **(a**–**c**) Micro-CT 3D reconstructed image of bone samples from normal (**a**), osteopenia (**b**), and osteoporosis (**c**) patients. (**d**–**i**) Quantification of the trabecular index of bone samples from normal (*N*=6), osteopenia (*N*=10), and osteoporosis (*N*=8) patients. (**j**–**l**) Bone immunostaining for CD31 (green), Endomucin (red), Osterix (gray), and DAPI (blue) for normal (**j**), osteopenia (**k**), and osteoporosis (**l**) patients. Graphs represent mean±S.D.; *P<0.05; **P<0.01; Scale bar: 100 *μ*m

The aging processes of bone and the loss of bone are closely related, and certain individuals lose bone density much faster than normal, which can lead to osteoporosis and an increased risk of fractures. Therefore, examined whether human type H vessels act as a sensitive marker for osteopenia or/and osteoporosis independent of aging. Patients were classified into three groups based on their bone mineral density (BMD) data according to WHO standards. Dual energy X-ray absorptiometry was performed for all patients to determine the patients’ BMD. Bone samples from normal (6 individuals), osteopenia (10 individuals), and osteoporotic (8 individuals) subjects were obtained from patients with hip fractures during PFN surgery. To evaluate the microarchitecture of the trabecular bone, a volume of interest that consisted of only trabecular bone was delineated in the *μ*CT image of each sample according to the recommendations of the American Society of Bone and Mineral Metabolism for calculating three-dimensional (3D) bone parameters and structural indices.^22^ The following parameters were named following the Parfitt system:^23^ bone volume (BV), total volume of interest (TV), bone volume fraction (BV/TV), trabecular thickness (Tb.Th), trabecular separation (Tb.Sp), trabecular number (Tb.N), and connectivity density (Conn.D). Quantitative measurements revealed that the BV/TV, Tb.Th, Tb.N, and Conn.D values were decreased with BMD (Supplementary Table S1) (*P*<0.05), which was consistent with a previous study.^24^ The 3D *μ*CT figures and quantification charts (Figures 3a–i) also showed that microarchitectural changes coincided with BMD. Thus, the patient groups were cross-calibrated by BMD and *μ*CT; however, significant differences were not observed between the groups according to age (*P*=0.065) (Supplementary Figure 3A and Supplementary Table S2). The other half of the human bone sample was subject to immunostaining to visualize type H vessels and osteoprogenitors. The percentage of HV/TV was 18.27±2.98% in the control and 12.48±1.28% in the osteopenia/osteoporosis subjects. We found that the abundance of type H vessels and osteoprogenitors was significantly reduced in the combination group of osteopenic and osteoporotic groups compared with that of the control group (*P*<0.05) (Figures 3j–l) (Supplementary Figure 3B). Interestingly, significant differences were not observed between the osteopenic and osteoporotic subjects (*P*=0.3893), although the effect was slightly less in osteoporotic subjects, indicating that a pronounced reduction of type H vessels occurred in the very early stage and was subsequently decelerated during bone loss and aging. Therefore, we propose that the abundance of type H vessels is an early sign of bone loss.

### Decreased type H vessels in an osteoporotic mouse model

**Figure 4:**
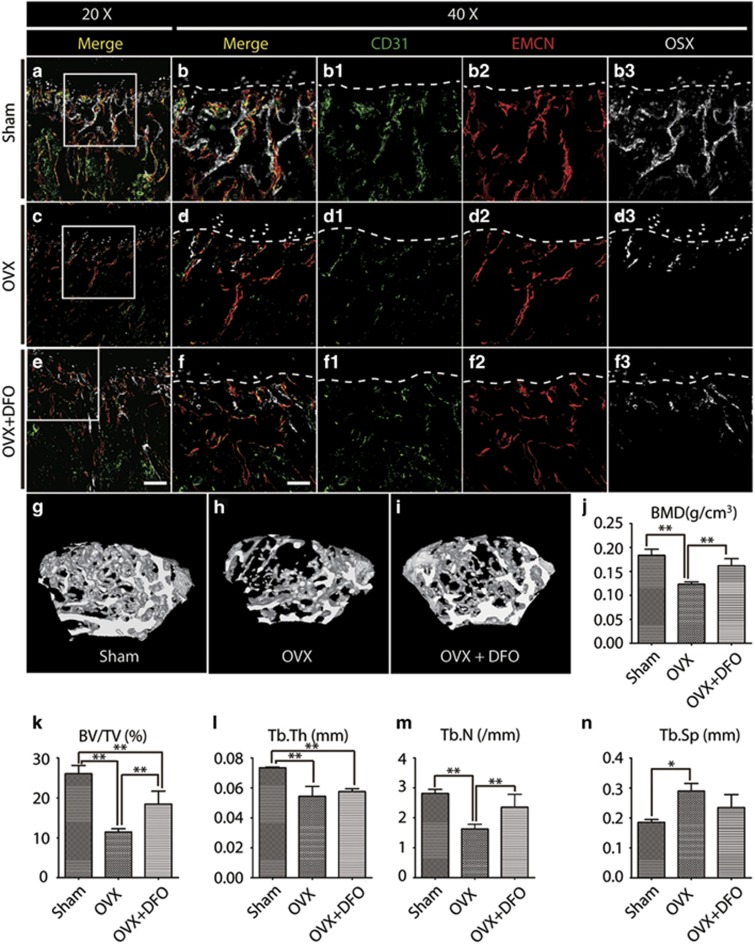
Change of type H vessels in osteoporotic mice. (**a**–**f**) Tibia immunostaining for CD31 (green), Endomucin (red), Osterix (gray), and DAPI (blue) of Sham (**a**–**b**), OVX (**c**–**d**), and OVX+DFO (**e**–**f**) group mice. Lines in **b**, **d**, and **f** indicate the boundary of the metaphysis and growth plate. (**g**–**i**) Micro-CT 3D reconstructed images of cancellous bone from the Sham (**g**), OVX (**h**), and OVX+DFO (**i**) mice. (**j**–**n**) Quantification of the BMD and trabecular index of bone from the Sham, OVX, and OVX+DFO mice. Graphs represent mean±S.D.; *P<0.05; **P<0.01; Scale bar: 200 *μ*m (**e)**, 100 *μ*m (**f**)

To further distinguish the underlying causes of the reduction in type H vessels associated with age or bone density or both, we established a typical osteoporosis model using young ovariectomized mice. As expected, both the BMD and trabecular indexes (BV/TV, Tb.N, Tb.Th, and Tb.Sp) exhibited significant changes in the ovariectomized group (Supplementary Table S3). Consistent with this finding, a histological analysis by hematoxylin and eosin staining also revealed that the trabecular indices decreased in the distal femurs of the ovariectomized mice compared with the sham-operated mice (Supplementary Figure 4). Because this process has been confirmed as representing osteoporosis, we next examined the expression of CD31, Emcn, and Osterix via immunostaining. We found that the abundance of type H vessels and osteoprogenitors was remarkably reduced in the metaphysis of ovariectomized mice (Figures 4c and d) compared with that of the sham-operated mice (Figures 4a and b), suggesting that type H vessels are a sensitive marker for bone density independent of age. To test whether enhanced angiogenesis can increase type H vessels and osteoprogenitors and therefore improve bone density, we treated the ovariectomized mice with DFO via intraperitoneal injection because DFO was previously reported to act as an angiogenic enhancing therapy to bolster vascular response in bone regeneration.^25^ DFO administration led to the partial rescue of type H vessels and an increase in osteoprogenitors at 4 weeks after injection (Figures 4e and f). More importantly, the increased type H vessels and osteoprogenitors were accompanied by microstructural changes verified by micro-CT data (Figures 4g–n) and hematoxylin and eosin staining (Supplementary Figures 4C, F and I). Together with our findings from osteopenic and osteoporotic subjects, type H vessels were significantly associated with bone quality. Therefore, we concluded that type H vessels are a highly valuable and sensitive biomarker of bone status and may be a potential target for bone loss therapies.

## Discussion

Different age groups and low bone density groups were evaluated, and the results demonstrated for the first time that the type H vessel is present in human bones and an abundance of human type H vessels represents a sensitive marker for aging and bone mass. In addition, we found that osteoprogenitor cells are enriched near type H vessels, and the DFO treatment increased the type H vessels and osteoprogenitors and improved the microstructure in mice. Thus, type H vessels have the potential for use as ideal therapeutic targets capable of simultaneously improving metabolic environments and delivering osteoprogenitors to prevent bone aging and osteoporosis and induce bone formation.

Recent reports have indicated that the new subtype of capillary endothelial cells referred to as type H vessels occur in the metaphysis and endosteum of murine long bone and provide a diagnostic target for the status of the bone vasculature and its pro-osteogenic capacity,^18, 26^ and these cells may also represent a novel potential therapeutic target for bone loss. Although the age-related loss of cancellous and cortical bone in mice is remarkably similar to age-related phenomena in humans,^27^ the remodeling processes, including the turnover pace, structures, and genetics, may differ between humans and mice. In humans, cortical bone is remodeled within the bone interior via teams of osteoclasts and osteoblasts that surround blood vessels, whereas in mice, the cortical bone directly contains the blood vessels.^28^ Thus, the establishment of type H vessels and their functional relevance in human bones are essential for further advancements. In the present experiment, because the sizes of the available samples were small and difficulties occurred during bone processing, we failed to obtain an intact organization of the skeletal vasculature in human cortical bone. Interestingly, we identified type H vessels in the proximal femur near the greater trochanter in human subjects. Moreover, the results indicating that human type H vessels are a sensitive and functional marker for aging and bone loss are particularly strong. In addition to demonstrating that CD31 and Emcn (Endomucin) are expressed in type H vessels in human bone, we also revealed that a reduced abundance of type H vessels is associated with advanced age in human subjects. Moreover, the reduction of type H vessels in the bone loss groups independent of aging may provide a therapeutic strategy involving enhanced angiogenesis for the prevention of bone loss. Finally, the subtle difference in type H vessels between osteopenia and osteoporosis demonstrates that changes in type H vessels represent an early event for bone deterioration. This finding is consistent with the response of type H vessels to certain signals, such as endothelial hypoxia-inducible factor (HIF) and the Notch pathway, in young mice.^18, 29^

Angiogenesis is crucial for osteogenesis as a functional vascular network and is key for bone development during embryo stage as well as bone remodeling thereafter.^30, 31, 32^ Furthermore, vascular endothelial cells bridge adjacent osteoblasts to accelerate healing during bone fracture.^33, 34^ In ovariectomized rat model, total vessel density was reported to be decreased together with bone mineral density (BMD) while treatment of dimethyloxalylglycine (a hypoxia-mimicking agent) prevented OVX-induced vessel loss and bone mass loss.^35^ In our study, we noticed that the type H vessel decreased more significantly along with bone mass loss in human and mice, providing a more specific target for future investigation. The effect of DFO on type H vessel in aged mice has already been discussed previously.^18, 26^ DFO inhibits prolyl-4-hydroxylases thus to enhance HIF-1*α* stability and activity, and therefore result in upregulation of angiogenic signals. Here in our study, DFO showed similar effect on OVX-induced osteoporosis and the mechanism is probably similar.

Aging and postmenopausal estrogen deficiency are major risk factors for osteoporosis characterized by abnormal bone metabolism, microarchitecture deterioration, and decreased bone strength, which can result in fragility fractures.^25, 26^ Although a number of intrinsic and extrinsic mechanisms are responsible for alterations in osteoblast number and function at the different stages of osteoblastogenesis, our data indicate that the angiogenic changes in postmenopausal osteoporosis are remarkable and may act as an indicator for the early stages of osteoporosis. The decreased functional capacity of the vascular system is likely to decrease the amount of oxygen in bone, impair the exchange of other nutrients, and lead to problems during the recruitment of osteoprogenitors to the site of bone formation. Multiple lines of evidence have suggested that estrogen directly modulates angiogenesis via its effects on endothelial cells.^36, 37^ However, estrogen treatment was found to increase bone density in ovariectomized rats by inhibiting bone resorption of osteoclasts accompanied by vascularization.^37, 38, 39^ Our data directly showed that type H vessels are surrounded by osteoprogenitor cells and present reduced numbers in osteoporosis subjects, and these findings have direct clinic relevance.

Most drugs currently used to treat osteoporosis are inhibitors of bone resorption and stabilizers of bone mass. However, these drugs were identified serendipitously and were not produced via rational drug design; therefore, they are not ideal because of their limited bioavailability and other unwanted effects. The discovery of type H vessels establishes a molecular framework that couples the activity of endothelial cells, chondrocytes, and osteoprogenitors in mice and human models. The development of technology to improve type H vessels in bone loss and bone healing represents a promising research path.

## Materials and Methods

### Patients

Patients admitted to our hospital with hip fractures were divided into three groups based on age and BMD. The age groups included patients from 20 to 35 years (young, 7 individuals), 45 to 59 years (adult, 8 individuals), and 60 to 75 years (aged, 24 individuals). Analyses regarding BMD consisted of all 24 samples from the aged patients. Twenty-four aged patients were divided into three groups according to T-scores.^40^ A T-score of ≥−1.0 was classified as normal; a T-score between >−2.5 and <−1.0 was classified as osteopenia; and a T-score of ≤−2.5 was classified as osteoporosis (Supplementary Table S2). All patients were enrolled if the following criteria were met: pertrochanteric fracture and indication of a surgical treatment. Patients who suffered from inflammation, metabolic endocrine diseases, and malignancy and those who received medicines affecting bone metabolism were excluded.

### Human bone samples

Human bone samples were collected as described in the ‘Results’ section. Briefly, samples were collected from patients who were admitted to our hospital with hip fractures and required a surgical intervention. Bone samples were collected during PFN for intertrochanteric femur fractures using a special instrument that is 6 mm in diameter. Fresh samples obtained during PFN were divided into two halves for micro-CT scanning and immunostaining. Iatrogenic bone loss did not occur from the surgery itself. Informed consent was obtained from each patient before the procedure, and this process was approved by the institutional review board of the Second Affiliated Hospital of Soochow University.

### Mice

C57BL/6 wild-type mice were used in this study. The animals were purchased from National Resource Center of Model Mice of Nanjing University and housed in the specific pathogen free barrier system with normal diet in the animal facility of Soochow University. Mice used in the age-series experiments were from 3 to 5 weeks (juvenile), 11 to 16 weeks (adult), and 47 to 52 weeks (aged). All mice-related works were performed in compliance with the relevant laws and internal guidelines of the Institutional Animal Care and Use Committee. All of the animal procedures were approved by the Animal Care and Use Committee of CAM-SU GRC, Soochow University.

### Osteoporotic mouse model

Eight-week-old female mice were randomly divided into three groups. All mice were anesthetized and subjected to bilateral ovariectomy (OVX) or a sham operation (sham). After a 1-week recovery period, the sham group mice were administered saline intraperitoneally (Sham group), whereas the OVX mice were administered saline (OVX group) or deferoxamine mesylate (DFO from Sigma, MO, USA, OVX+DFO group). For the DFO treatment, freshly prepared DFO in 0.9% saline (250 mg/kg body weight) was administered every other day for 4 weeks. The mouse tissues were collected at week 4 to investigate the vascular and osseous changes via immunostaining, hematoxylin and eosin staining, and micro-CT scanning.

### Micro-CT analysis

Mouse femora were dissected at 4-week time points, and the attached soft tissue was completely removed. Fresh human bone samples were obtained during surgery and then fixed in 4% paraformaldehyde and analyzed by micro-CT (Skyscan 1176 *In Vivo* Micro-CT, BRU KER, Kontich, Belgium). The scanners were set at the voltage of 50 kV, a current of 500 *μ*A, and a resolution of 9 *μ*m for the mouse femora samples and at 65 kV, 385 *μ*A, and 18 *μ*m for the human bone samples. Image software (NRecon v1.6) and data analysis software (CTAn v1.13.11.0 and 1.11.10.0) were used for the 3D reconstruction of the trabecular bone.

After scanning, a constant region of interest was defined by the analysis software. Then, 3D images of the trabecular bone were reconstructed based on the region of interest. In the human bone samples, the region of interest was outlined by a rectangular box-sized 2.9 mm × 2.9 mm × 2.78 mm, whereas in the mouse femoral samples, the region of interest was outlined starting from a point 540 *μ*m proximal to the distal growth plate and extending 1.35 mm toward the diaphysis. The following parameters of the trabecular bone were calculated:^22, 41^ bone mineral density (BMD; g/cm^3^), bone volume/tissue volume (BV/TV; %), trabecular thickness (Tb.Th; mm), trabecular number (Tb.N; /mm), trabecular separation (Tb.Sp; mm), structure model index (SMI), and connectivity density (Conn.D; /mm^3^).

### Antibodies

Rabbit-anti-mouse Osterix (OSX) (Santa Cruz, Dallas, TX, USA), rat-anti-mouse Endomucin (EMCN) (Santa Cruz), Alexa Fluor 488-conjugated anti-mouse CD31 (R&D, Minneapolis, MN, USA), Alexa488-mouse-anti-human CD31 (Dako, Carpinteria, CA, USA), rat-anti-human Endomucin (abcam, Cambridge, MA, USA), Cy3- or Alexa647-conjugated secondary antibodies (Molecular Probes, Eugene, OR, USA) were used.

### Bone tissue processing and immunostaining

Freshly collected specimens were fixed in 4% paraformaldehyde overnight at 4 °C, and EDTA solution was then applied for decalcification for at least 2 weeks. The tissues were then dehydrated using 20% sucrose and 2% polyvinylpyrrolidone solution for 1 week at 4 °C and then embedded in O.C.T. compound (Tissue-Tek, Sakura Finetek, Torrance, CA, USA). Cryosections (10 *μ*m) were prepared using freezing microtome (Leica, Wetzlar, Germany) for immunostaining.

For immunostaining, the bone sections were washed three times with 0.3% PBST (Triton X-100) and blocked with 5% bovine serum albumin in PBS. Sections were incubated with primary antibodies at 4 °C overnight. After three washes with 0.3% PBST, the sections were then incubated with fluorescein-conjugated secondary antibodies together with nuclear counterstaining dye (DAPI) at room temperature. After another three washes, slides were mounted with 50% glycerol and visualized under a confocal microscope (Olympus, Tokyo, Japan).

### Quantification of blood vessels

To quantify the blood vessel area in the stained sections, at least three images (with × 200 magnification) representing different fields were obtained using a confocal microscope. The blood vessel area was measured using Image-Pro Plus (Media Cybernetics, Rockville, MD, USA) based on color recognition.

### Statistical analysis

All data were expressed as the mean±S.D. Statistical analyses were performed using a one-way analysis of variance (ANOVA) with Student–Newman–Keuls post hoc test for multiple comparisons. *P*<0.05 was considered statistically significant and indicated by ‘*’ *P*<0.01 was indicated by ‘**’ *P*<0.0001 was indicated by ‘***’. All statistical analyses were performed using SAS 8.2 software.
