# Supplementary material for: Human type H vessels are a sensitive biomarker of bone mass
Source: Cell Death Dis. 2017 May 4;8(5):e2760–. doi: 10.1038/cddis.2017.36 (PMC5520742; doi:10.1038/cddis.2017.36)
Supplement: Supplementary Information [file cddis201736x1.docx]

**Supplementary Materials:**


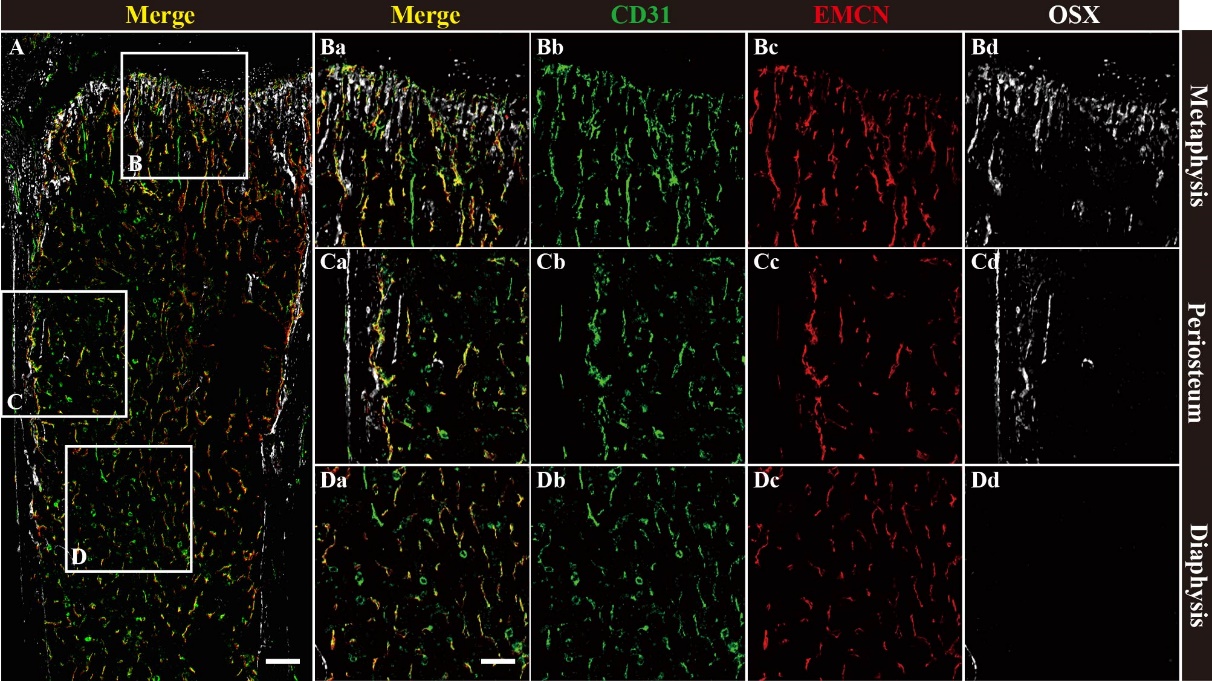


**Supplemental Fig. 1. Immunostaining of 4-week mouse tibia.** Immunostained mouse tibia was imaged under a confocal microscope to capture different fields representing different regions of tibia, including the metaphysis (**B**), periosteum (**C**) and diaphysis (**D**). Staining antibodies: CD31 (green), Endomucin (red) and Osterix (gray). Scale bar: 200 μm in **A**, 100 μm in **D**.


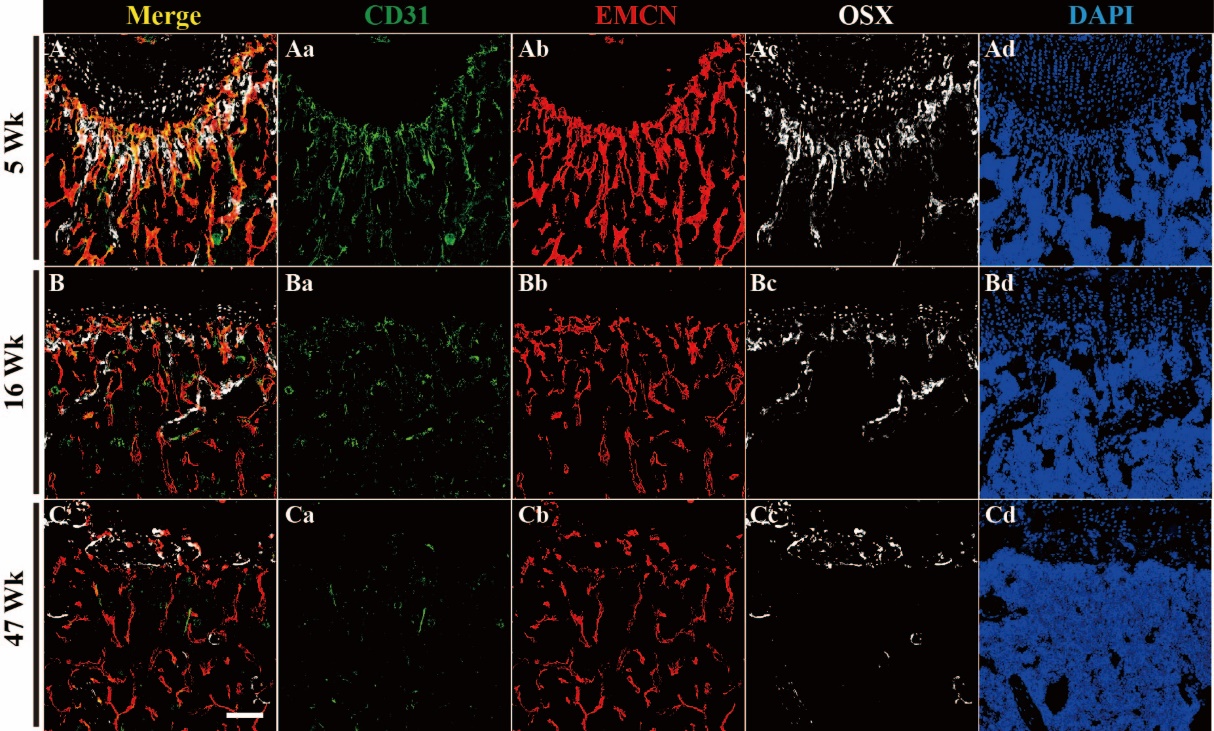


**Supplemental Fig. 2. Immunostaining of distal femur.** Distal femora from juvenile (5 weeks, **A**), adult (16 weeks, **B**) and aged (47 weeks, **C**) mice were stained for CD31 (green), Endomucin (red), Osterix (gray) and DAPI (blue). Scale bar: 100 μm.


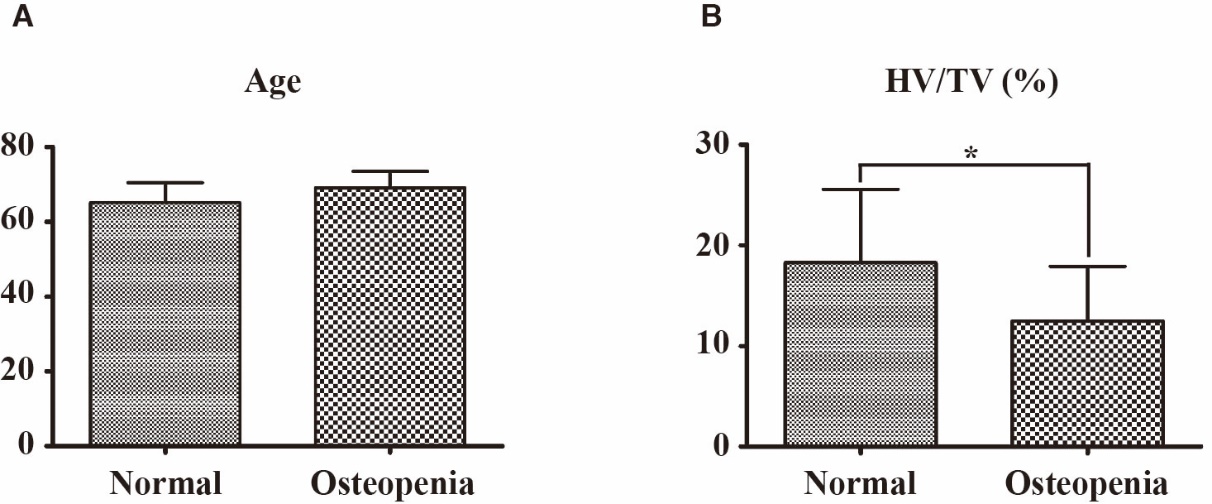


**Supplemental Fig. 3. Type H vessel in aged patients. A**, Difference of age in normal and osteopenia/osteoporosis groups. **B**, Quantification of type H vessel in normal and osteopenia/osteoporosis groups. *: P<0.05.


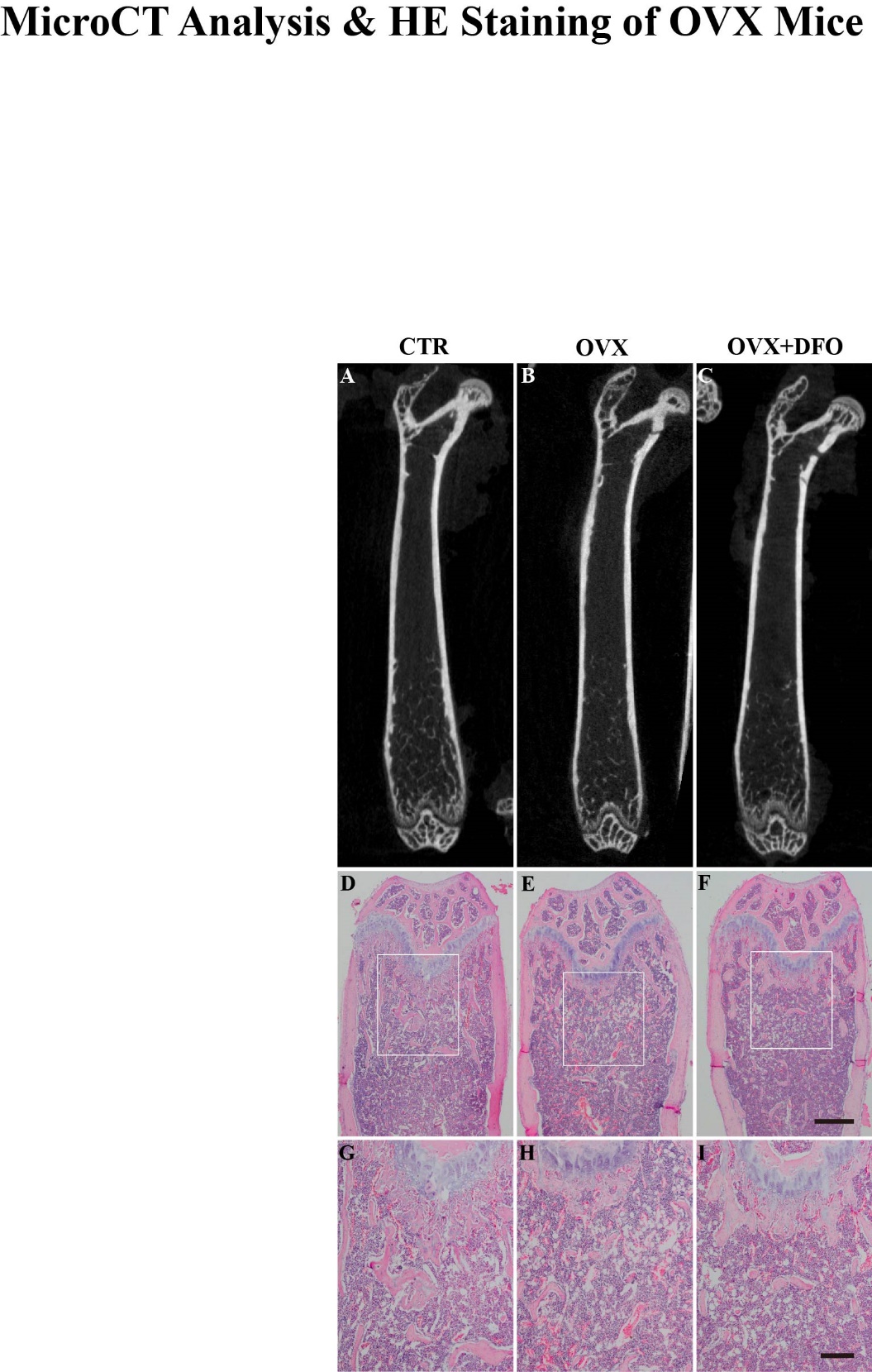


**Supplemental Fig. 4. Micro-CT and histological images of distal femur.** **A-C**, Micro-CT images of the Sham (**A**), OVX (**B**) and OVX+DFO (**C**) treated mice demonstrate that DFO partly rescued the deterioration of the bone microarchitecture induced by OVX. **D-I**, HE staining of the distal femur demonstrates that changes of trabecular under the growth plate in the Sham (**D** and **G**), OVX (**E** and **H**) and OVX+DFO (**F** and **I**) treated mice. Scale bar: 500 μm in **F**, 200 μm in **I**.

**Table S1. Micro-CT analysis of bone microarchitecture from patients**

|  | Normal | Osteopenia | Osteoporosis |
| --- | --- | --- | --- |
| BV/TV(%) | 24.93±7.72^#^ | 14.28±1.65^*^ | 10.47±1.44^*^ |
| SMI | 1.96±0.27^#^ | 1.86±0.21^#^ | 2.36±0.10^*^ |
| Tb.Th(mm) | 0.24±0.03^#^ | 0.19±0.03^*^ | 0.17±0.01^*^ |
| Tb.N(/mm) | 1.05±0.29^#^ | 0.75±0.06 | 0.60±0.11^*^ |
| Tb.Sp(mm) | 0.59±0.09 | 0.74±0.11 | 0.74±0.09 |
| Conn.D(/mm^3^) | 5.85±3.85 | 1.37±0.65 | 2.00±0.72 |

The groups designated with an asterisk exhibit significant differences with normal group, the groups designated with a pound sign exhibit significant differences with osteoporosis group. BV/TV, bone volume fraction; SMI, Structure model index; Tb.Th, Trabecular thickness; Tb.N, Trabecular number; Tb.Sp, Trabecular separation; Conn.D, Connectivity density.

**Table S2. Clinical data of human in age and BMD groups**

| **Group** | | **AD** | **Age** | **T-F** | **T-L** | **HV/BV (%)** |
| --- | --- | --- | --- | --- | --- | --- |
| Young | | 520082 | 33 | 1.5 | 2 | 31.22±2.79 |
|  |  | 531642 | 20 | 1.6 | 1.1 | 30.46±14.42 |
|  |  | 530180 | 29 | 0.2 | -0.7 | 35.19±7.31 |
|  |  | 528998 | 31 | 1.8 | 0.9 | 31.07±5.18 |
|  |  | 531162 | 21 | -0.9 | -0.8 | 35.26±3.45 |
|  |  | 531370 | 24 | 0.2 | 1.1 | 32.65±1.44 |
|  |  | 518355 | 28 | -0.1 | 0.9 | 31.39±3.92 |
| Medium | | 522073 | 48 | 0.4 | 0.3 | 19.33±3.13 |
|  |  | 522041 | 54 | 0.6 | 0.8 | 21.30±6.39 |
|  |  | 510741 | 47 | 0.8 | 0.1 | 19.45±3.48 |
|  |  | 526175 | 57 | 0.7 | -0.1 | 16.86±2.33 |
|  |  | 517586 | 43 | 1.1 | 0.2 | 17.20±1.34 |
|  |  | 522073 | 48 | 0.4 | 0.3 | 17.66±0.86 |
|  |  | 523811 | 55 | -1 | 0.2 | 19.38±2.11 |
|  |  | 524825 | 53 | -0.2 | -0.6 | 20.22±2.28 |
| Aged | Normal | 513373 | 62 | -0.2 | -0.4 | 22.43±6.45 |
|  |  | 501402 | 61 | 0.5 | 1.2 | 14.30±2.38 |
|  |  | 548389 | 65 | -0.6 | -0.2 | 28.64±2.26 |
|  |  | 502623 | 60 | 0.5 | 0.8 | 10.89±1.73 |
|  |  | 514117 | 74 | -0.3 | -1 | 22.44±1.41 |
|  |  | 539199 | 71 | -1 | -1 | 10.93±1.31 |
|  | Osteopenia | 503141 | 75 | -1.2 | -0.4 | 10.00±1.96 |
|  |  | 521512 | 63 | -1.7 | -2.2 | 16.23±1.01 |
|  |  | 548806 | 75 | -2.2 | -2.3 | 14.27±1.52 |
|  |  | 551763 | 71 | -1.3 | -1.6 | 16.60±1.58 |
|  |  | 452585 | 67 | -1.4 | -2.2 | 23.43±6.30 |
|  |  | 503506 | 68 | -1.5 | -1.3 | 11.07±2.47 |
|  |  | 504454 | 73 | -1.3 | -1.5 | 8.52±2.01 |
|  |  | 503450 | 65 | -1.3 | -0.7 | 11.25±1.64 |
|  |  | 520357 | 67 | -1 | -1.1 | 10.33±2.04 |
|  |  | 552713 | 60 | -1.2 | -2.3 | 13.29±4.44 |
|  | Osteoporosis | 521993 | 66 | -1.6 | -2.8 | 13.72±2.03 |
|  |  | 453073 | 71 | -2.4 | -4.1 | 7.06±4.67 |
|  |  | 469810 | 68 | -2 | -2.9 | 20.06±1.69 |
|  |  | 482765 | 66 | -2.1 | -2.8 | 21.94±5.77 |
|  |  | 497063 | 73 | -2.7 | -3.4 | 6.15±3.29 |
|  |  | 499559 | 71 | -3.1 | -2.3 | 5.94±3.90 |
|  |  | 562945 | 63 | -3.1 | -1.1 | 8.56±2.73 |
|  |  | 524682 | 73 | -2.7 | -3.8 | 6.25±1.47 |

AD, admission number; T-F, femoral neck T-scores; T-L, lumbar T-scores; HV, type H vessel; BV, blood vessel

**Table S3. Micro-CT** **analysis of BMD and key parameters of trabecular bone in mouse**

|  | Sham | OVX | OVX+DFO |
| --- | --- | --- | --- |
| BMD(g/cm^2^) | 0.18±0.01^#^ | 0.12±0.00^*^ | 0.16±0.01^#^ |
| BV/TV(%) | 26.05±2.05^#^ | 11.47±0.79^*^ | 18.44±3.26^*#^ |
| Tb.Th(mm) | 0.07±0.00^#^ | 0.05±0.01^*^ | 0.06±0.00^*^ |
| Tb.N(/mm) | 2.81±0.13^#^ | 1.63±0.15^*^ | 2.36±0.43^#^ |
| Tb.Sp(mm) | 0.19±0.01^#^ | 0.29±0.03^*^ | 0.24±0.04 |

The groups designated with an asterisk exhibit significant differences with Sham group, the groups designated with a pound sign exhibit significant differences with OVX group. BMD, bone mineral density; BV/TV, bone volume fraction; Tb.Th, Trabecular thickness; Tb.N, Trabecular number; Tb.Sp, Trabecular separation.
